# Editorial: Women in psychiatry 2023: ADHD

**DOI:** 10.3389/fpsyt.2024.1447958

**Published:** 2024-08-14

**Authors:** Annet Bluschke, Noemi Faedda, Julia Friedrich, Eleanor J. Dommett

**Affiliations:** ^1^ Department of Child and Adolescent Psychiatry, University Hospital and Medical Faculty Carl Gustav Carus, Technische Universität Dresden, Dresden, Germany; ^2^ Department of Human Neuroscience, Sapienza University of Rome, Rome, Italy; ^3^ Institute of Systems Motor Science, University of Lübeck, Lübeck, Germany; ^4^ Department of Psychology, Institute of Psychiatry, Psychology and Neuroscience, King’s College London, London, United Kingdom

**Keywords:** ADHD, impact, gender, parents, treatment

ADHD is a common neurodevelopmental condition affecting all ages (1, 2). It is associated with elevated risk of various co-occurring conditions, educational, occupational and social difficulties (3). In the last two decades recognition and treatment of ADHD has increased (4, 5) resulting in long waiting lists and treatment delays, making ADHD an unmet health need (6). Within this context, gender inequalities also exist with women waiting four years longer for diagnosis (7) and calls for research to consider female reproductive hormones (8). One factor that may contribute to this inequality is low representation of females in ADHD research (9). It is therefore timely that this Research Topic showcases research by women into ADHD.

## Understanding the impact of ADHD

The impact of ADHD is explored in the review by French et al. who summarize three key risk domains: mental health, physical health, social and lifestyle factors. Mental health outcomes consistently associated with ADHD include increased rates of addictions, self-harm and suicidality, psychiatric and personality disorders and poor self-esteem. The authors suggested that emotional dysregulation may contribute to the overlapping conditions along with impulsivity, which may play a role in completed suicide and self-harm. However, they emphasized that further research is needed to determine whether outcomes associated with poor mental health arise as comorbidities which only emerge at specific points in life or whether ADHD is a precursor to and risk factor for poor mental health. Physical health outcomes associated with ADHD include poor sleep and oral health, obesity and higher risk of accidents and injuries, and various diseases, further demonstrating the burden experienced beyond the direct effects of ADHD. French et al. noted that the research for sleep lacked use of objective measures and that there was potential for a vicious circle to be created where poor sleep increased ADHD symptom expression and how improving sleep might benefit core symptoms. The poor oral health in ADHD was associated with the core symptoms of the condition, because those with ADHD are more impulsive and impatient which may lead to poor teeth cleaning, along with consumption of more sugary food. Consumption of sugary food was not, however, linked to obesity in ADHD which appeared to be mediated by poverty, although the authors noted a need to develop more understanding of how the core symptoms of ADHD impact eating habits. The review outlined the type of accidents and injuries that typically occur but did not link these specifically to the core ADHD symptoms, although one could speculate that these play a role e.g. inattention and impulsivity may increase the risk of car accidents, which are greater in adults with ADHD. In terms of the diseases, a range of different conditions were identified as associated with ADHD including migraine and chronic pain. Mechanisms were not explored. For lifestyle and social factors strong links were evident between ADHD and criminality, poor educational attainment, relationship difficulties, and risky behaviours like driving-related incidents. For criminality, the authors reported that several reviews noted that ADHD was associated with criminal behaviour, offending and incarceration, but there was also a greater risk of those with ADHD being the victim of intimate partner and sexual violence. It is suggested that criminal activities reported are likely to have an impulsivity component, linking to the core symptoms of ADHD, and that different comorbid conditions such as Conduct Disorder may also play a role, suggesting that effectively treating these symptoms and comorbidities would reduce criminal outcomes. The authors noted that poor educational and occupational outcomes were associated with ADHD, especially when it was left untreated, and made a call for urgent research to be conducted into what support was effective for children with ADHD at school and adults in the workplace. The authors suggested that the difficulties in social relationships (peer and intimate) may be partially mediated by comorbid conditions such as Conduct Disorder which could result in social cognition difficulties. The risk-taking behaviours reviewed included those that linked to the early section on accidents and injuries e.g. driving outcomes, but this section included generally lower quality reviews, resulting in the authors emphasizing the need for further research and consideration of co-morbidities. Perhaps unsurprisingly given the various associations identified, ADHD was also consistently linked to reduced quality of life. In summary, this review emphasizes the wide implications of ADHD and the need for whole-person treatment approaches, which consider co-occurring conditions and tailored interventions. By identifying these risks and impairments, French et al. lay the foundations for future research to improve the negative outcomes associated with ADHD.

## Improving understanding: tools to enhance clinical practice

Two studies in this topic have the potential help further understand ADHD and inform clinical practice. Firstly, Kochhar et al. address a key challenge in differential diagnosis, specifically distinguishing between ADHD and Autism Spectrum Disorders (ASD), using visual attention to social stimuli. They report that children with ASD (with or without comorbid ADHD) show reduced fixation to faces compared to children with ADHD alone. In addition, this fixation duration negatively correlated with severity of communication and repetitive stereotypy symptoms. This suggests that assessing visual attention to social cues might assist clinicians when considering diagnosis of ADHD and ASD. Secondly, Skliarova et al. explore self-efficacy in ADHD; a psychological protective factor thought to be reduced in the condition. Examining the applicability of a shortened version of the General Self-Efficacy scale in adults with ADHD, they report satisfactory content validity and good psychometric properties as well as positive correlations between self-efficacy and general well-being. This research offers the possibility of easily evaluating self-efficacy in adult ADHD, which could provide insights into mental health and well-being.

## Managing ADHD: parents, experience, efficacy, and personalisation

The importance of multidisciplinary treatment, including parental interventions, is widely recognised (10). Behavioural parent training (BPT) provides parenting strategies to promote desirable behaviours and minimise unwanted ones in children with ADHD. Despite effectiveness, access to BPT is limited (11). Bado et al. conducted a needs assessment to understand the experiences and treatment needs of families with children showing ADHD symptoms in Brazil. Semi-structured interviews with parents, educators, and healthcare providers revealed several themes: parents often reported minimal involvement in their child’s psychotherapy; a few parents learned behavioural management strategies from healthcare providers; many parents desired practical information on managing their children’s behaviours daily and managing their stress when children did not follow directions. Furthermore, some parents and professionals suggested families would benefit from learning more about ADHD and practical parenting strategies. A second article on parenting emphasizes how ADHD symptoms in adults could interfere with parental functioning. In this paper Miklósi et al. highlight the importance of parental cognitions on child development and present a meta-analysis of 15 high-quality studies exploring the relationship between parental ADHD symptoms and parental cognitions. They found that parents with higher ADHD symptoms reported more negative cognitions. Their results suggested that stressful childrearing may trigger dysfunctional cognitions which results in a negative perception of the parental role, the child and co-parenting. Repeated parenting difficulties can then exacerbate parental stress and negatively impact parent-child relations. Addressing dysfunctional parental cognitions is therefore crucial in parents with ADHD symptoms. This work also highlights the need for multi-method, multi-informant research to better understand and support parents with ADHD.

Two articles focus on different approaches to managing ADHD. William et al. investigate Cognitive Behavioural Therapy (CBT), a recommended and well-established approach (12). They present mixed methods research incorporating a survey and interviews, which were thematically analysed to show that individuals with ADHD may find CBT a negative experience when it is not tailored to ADHD, creating an unhelpful and overwhelming experience. Guimarães et al. examine the novel approach of transcranial direct current stimulation (tDCS) for ADHD, an approach that has shown some promise in preliminary research. In a triple blind study, they found no improvement in attention, working memory or response inhibition. The authors acknowledge limitations of the work, but the robust design presents a convincing argument for ineffectiveness.

The final two studies in this topic speak to the aforementioned gender inequality. Firstly, de Jong et al. present a case study exploring the effects of adjusting stimulant medication in the premenstrual week. Building on research indicating that stimulants are less effective in the luteal phase, they increased medication for nine women with ADHD by 41% on average. Women reported improvements which brought this week into alignment with others for symptom experience. The authors call for further work investigating this and consideration of hormones more broadly to support a personalised approach. Secondly, work by Praus et al. examined responsiveness to telemedicine, an approach that could reduce the crisis in ADHD care. They examined the characteristics of those who responded differently to this approach and revealed that those with higher depression scores, females and those living with children had a poorer outcome, again reinforcing the need to consider individual factors to optimise treatment. All articles in this topic point to the need for more research to ensure a deeper understanding of, and appropriate personalised management for, ADHD.
